# How do clients in Australia experience Consumer Directed Care?

**DOI:** 10.1186/s12877-018-0838-8

**Published:** 2018-06-26

**Authors:** Liz Gill, Sandra L. Bradley, Ian D. Cameron, Julie Ratcliffe

**Affiliations:** 1 John Walsh Centre for Rehabilitation Research, Sydney Medical School Northern, The University of Sydney, Sydney, NSW Australia; 2 College of Nursing and Health Sciences, Flinders University, GPO Box 2100, Adelaide, SA 5001 Australia; 3 Institute for Choice, UniSA Business School, Way Lee Building, Room WL3-65, City West Campus, Adelaide, South Australia Australia

**Keywords:** Consumer directed care, Clients, Australia, Thematic, Community

## Abstract

**Background:**

Our study explored client experience of Australian Consumer Directed Care. This evolving funding model enables consumer autonomy and choice, allowing older people to remain in their community as they age and need support through the creation of a personalised support service. Consumer Directed Care focuses on providing services that the consumer self-determines to meeting their needs including identifying their types of services, from whom, when and how these services are delivered.

**Methods:**

Semi-structured in-depth interviews were conducted in two Australian states between August 2015 and April 2016 with 14 participants, preferably in receipt of CDC services for at least the previous 12 months. Questions explored how the participant first learned about this service; the types of services they received; whether services met their needs; and any additional support services they personally purchased. Interviews were transcribed, coded and thematically analysed.

**Results:**

Four main themes related to consumer experience emerged. *Knowledge*: Unsure what Consumer Directed Care Means. *Acceptance:* Happily taking any prescriptive service that is offered. *Compliance*: Unhappily acceding to the prescriptive service that is offered. *External Influences:* Previous aged care service experience, financial position, and cultural differences.

**Conclusion:**

Our results suggest that the anticipated outcomes of Consumer Directed Care providing a better service experience were limited by existing client knowledge of these services, how best to utilise their funding allocation, and their acceptance or compliance with what was offered, even if this was not personalised or sufficient. External influences, such as service experience, finances, cultural difference, impacted the way clients managed their allocation. Our study identified that ongoing engagement and discussion with the client is required to ensure that services are specific, directly relevant and effective to achieving a consumer directed care service.

**Electronic supplementary material:**

The online version of this article (10.1186/s12877-018-0838-8) contains supplementary material, which is available to authorized users.

## Background

In Australia, provision of services to older people who meet specific requirements for home care continues to evolve as consumers and service providers, including government, negotiate growing demand and what is required to enable the consumer to manage their own lives in their own homes with support how and as they need it [1, 2]. The Consumer Directed Care (CDC) approach has been adopted internationally to promote consumer autonomy and choice so older people can live in a community setting when they require assistance with their activities of daily living [3–6]. According to the Australian Government Department of Health [6], “Consumer Directed Care (CDC) is both a philosophy and an orientation to service delivery and planning of care”. The main objective of CDC is to offer consumers more choice and flexibility about the types of care and services they receive, how they are delivered, by whom and when [6, 7]. It is anticipated that this will improve the individual’s health and quality of life (QOL) such that entry into residential aged care or hospital is delayed or minimised and the person remains in the place of their choice, usually their home, whilst receiving appropriate support services.

Historically, the current CDC model in Australia arose as part of the recommendations of the Australian Government Productivity Commission Report on Caring for Older Australians [8] which led to a ten-year program of reform called *Living Longer, Living Better,* due for completion in 2022 [9]. These reforms were acknowledged as needed because of the demographic bulge of the Baby Boomers entering older age [9]. There have always been home care packages subsidised by the government for those in need but these have been delivered through care providers who had full control of actual service provision and delivery [2, 8–10]. In 2012–2013, the first group of new home care packages (around 5800) based on CDC were allocated on a trial basis [9]. From that time, evaluations have been conducted on how consumers and providers perceive this new direction in care [1, 11, 12]. Based on results of these trials, from July 2015, all new aged care packages have been required to operate on a CDC basis and previous package models have been transitioning across to CDC. In 2018 all home care packages are required to be of the CDC type [10].

Whilst there is no single definition or model of CDC, the distinguishing feature of this type of service relates to the client or their representative having “control” over their allocated funds to meet the individual’s specific needs [11]. In Australia, control is now through the consumer directing their budget and services. However prior to CDC, home aged care packages were managed by approved providers on the basis of their delivering a set quantity of packages within specific geographic areas [12]. With the introduction of CDC, past providers have had to adapt, yet there are still major waiting lists (especially for level 3 and 4 packages) and the funding does not go far enough due the significant administrative costs being charged and the wide use of brokering [13]. To improve the capacity of the package to provide the care required on an individual basis, the Australian Government informed providers that “Flexibility and choice are the foundations of CDC. You have the ability to broker out services to meet the needs of your clients if you are not able to provide a specific service yourself” [14].

At the time our study was conducted (August 2015 to April 2016), when full funding conversion to the CDC model had not taken place. Notably, CDC was to be rolled out in two more stages with Stage 1 having commenced in February 2017 [10], where funding for a home care package became tied to the individual consumer (rather than the provider), and Stage 2 commencing in July 2018, where the Home Care Packages Program and the Commonwealth Home Support Program would become integrated into a single program, simplifying the way that services are delivered and funded [15].

These staged processes have been introduced in response to the findings of the Productivity Commission 2011 inquiry [8] which found that the aged care system suffered key weaknesses, including a high regulatory burden, lack of timely access to care, and limited consumer choice [16]. However in Australia, there has been little substantial research conducted into CDC within the aged care sector [17] with few national or international studies identifying specific choices made and specific outcomes achieved by CDC clients [18]. Yet, Bowers et al. [19] argue that models of care delivery such as CDC must be based on mutuality and/or reciprocity if older people with high support needs are to live well in later life. Given that the resource implications and costs associated with CDC can be substantial, it has been argued [3, 20, 21] that it is important to specifically identify the health, well-being and quality of life (QOL) gains associated with CDC use compared to Provider Directed Care services (PDC).

To provide more depth of information on the experience of receiving consumer directed care, a four phase Consumer Directed Care (CDC) research project was collaboratively conducted by Flinders University in South Australia (SA) and the University of Sydney in New South Wales (NSW) [22]. Funded by an Australian Research Council Linkage Project grant (LP110200079), the study used a steering committee that included representatives of five CDC service providers (Helping Hand SA, ACH Group SA, Resthaven Incorporated SA, HammondCare NSW, and Catholic Community Services NSW). Ethics approval for the research project was obtained from the Ethics Committees of Flinders University and the University of Sydney, along with approval from the five partner organisations. To ensure participant confidentiality and privacy, the service organisations were not informed which of their clients were being interviewed for the study.

This paper describes the findings of a second qualitative investigation which examined client experience of the CDC rollout. The first qualitative investigation was undertaken between December 2012 and November 2013, prior to the main CDC roll out during the early pilot phase and involved qualitative interviews with older people to determine their knowledge and attitudes towards CDC in community aged care [11]. Interviews were conducted with older people to specifically investigate their experience with the model. This included the types of services they chose; how they interpreted the information provided to them; any barriers or facilitators to their understanding and use of CDC; and any additional services that they paid for separately because of changes to their previous home care package or limitations of the CDC package.

## Methods

This study constituted part of the final phase of the CDC research project. The first phase of the project was completed in 2014 and involved the first qualitative study [11]. The second phase was completed in 2015 and involved the development and administration of a discrete choice experiment to quantify the preferences of older people for salient attributes defining the CDC model of service delivery [23]. The third stage of the project comprised an in-depth evaluation of the costs and consequences of CDC measured by health and quality of life outcomes [24].

To recruit participants, this current research study was described to clients by their service provider (manager or frontline worker) who, if the client expressed an interest in the study, provided the client with a copy of both the Letter of Introduction and an Information Sheet about the study. If the client then indicated a willingness to participate in the study, the client was asked by their care worker to complete the Consent Form and the completed Consent Form along with the participant’s name and details was provided to a researcher in the state in which it applied (LG for New South Wales; SB for South Australia). The state-based researcher then contacted the client, described the study in more detail, and if they were still agreeable, made arrangements for an interview at a time and location of their choice. It was reiterated throughout this process that they could withdraw from the interview and the study at any time without prejudice and could refuse to answer any specific question they were not comfortable answering. To ensure participant confidentiality and privacy, the service organisations were not informed which of their clients were being interviewed for the study.

Eligibility criteria for participants were: in receipt of a CDC service for the previous 12-month period; not in receipt of any other type of community aged care service other than CDC; and had experienced CDC only (not previous models of aged care). No limitation was placed on participation by age, gender, package level, location (other than state-based) or service provider. Participants had to have enough cognitive ability to be able to provide informed consent.

As the study commenced, it was discovered that because the study was undertaken during the period of transition from PDC to CDC it was not possible to recruit solely those who had only experienced CDC. Therefore, the recruitment pool was expanded to include clients who had transferred from PDC to CDC. In addition, in three instances a primary caregiver was present at the time of interview without prior knowledge of the researcher. In these cases, consent from both parties was obtained prior to the interview being conducted with participation encouraged from the client as much as possible, rather than the carer, as it was found that all clients had capacity to understand and answer questions asked.

Semi-structured interviews were conducted between August 2015 and April 2016 with questions (Additional file 1) designed to elicit information specifically about:

1. how the participant first learned about CDC,
2. the types of services they received,
3. whether the services met their needs and
4. whether they were paying for other services not covered by their existing CDC allocation.

These questions were derived from issues identified in the previous research phases which have since been published [11, 20]. Questions were designed to explore multiple facets of CDC implementation including the sufficiency of the client’s budget and the procurement of additional services. In this fourth phase, questions delved deeper into the actual lived experience of receiving a CDC package. All interviews were audiotaped and sent to a commercial third-party transcription agency.

Post-interview field notes were constructed after each interview to record the researcher’s observations, feelings and impressions, and identify any personal assumptions, actions and interpretations that had occurred because of the research process. After LG and SB had each conducted one interview, both researchers’ interview data and field notes were compared to assess whether additional further exploratory questions should be asked, for example, participants did not seem to know the term “CDC” so a question was framed to generate participant understanding of the terminology surrounding CDC. This process continued for each interview until the researchers agreed that no new topics were being raised that required further exploration and data saturation had been achieved.

Interviews and thematic analysis were conducted by LG and SB using NVivo 10 according to classical grounded theory guidelines [25]. For thematic analysis, each interview was initially coded by single words or phrases that described a concept raised by the participant in relation to the CDC package, for example “pets” in relation to paying for the care of a pet if the person went to hospital (Additional file 2). From the first interview, these words or phrases were explored in subsequent interviews to extend meanings. Once coding of all the interviews was completed, categories were created through combining codes with similar ideas. These categories were then further summated and themes created that articulated more fully the concepts being expressed. Both LG and SB discussed the emerging categories and themes until achieving consensus that the themes adequately reflected the perspectives of the participants. Once themes were generated, authors JR and IC provided further review and consideration of the findings. A draft of the final paper was presented to a representative from the service providers for comment prior to the submission for publication. Representative input provided clarity to the way service providers could offer CDC packages but did not change the articulation of the consumer perspective.

## Results

Initially, the five service provider organisations identified a total of 25 potential participants for the study. Of the 25, only 14 agreed to participate with those declining doing so due to either ill health, not enough time, or experiencing an acute health event. Six of the 14 participants were men and eight were women with an age range from 65 to 98 years and a mean age of 82.5 years. Five participants were first time recipients of aged care services while nine had been transferred from a PDC package to a CDC package. All participants resided in urban areas. Services described by participants included: cleaning, shopping, transport, bill paying, companionship, personal grooming, toileting, medication management, respite and entertainment.

From the 14 interviews conducted, 65 initial codes were created (Additional file 2). Examples of these codes include:

- “house maintenance” such as gutter cleaning or window repair, as opposed to house cleaning services
- “terminology” wherein the participant explains their understanding of some of the CDC terms, their knowledge or lack of understanding/knowledge of the meaning of CDC
- ‘just told’ which exemplifies the manner in which information was conveyed to them
- “incontinence” as an example of specific health conditions influencing the type of service required
- “paying bills” as a way of describing financial considerations undertaken when choosing different CDC or non-CDC services.

The 65 codes were then combined into 9 categories representing the overall CDC experience from the client perspective (Additional file 2). Examples of these categories include:

- getting information rather than knowledge
- vulnerability to service reduction
- experience with previous aged care packages
- the assessment process

From these 9 categories, four main themes emerged (Additional file 2):

- *Knowledge: Unsure what Consumer Directed Care Means*
- *Acceptance: Happily taking any prescriptive service that is offered*
- *Compliance: Unhappily acceding to the prescriptive service that is offered*
- *External Influences: Previous aged care service experience, money and culture*

These four themes are described in more detail in the sections that follow.

### Knowledge: unsure what consumer directed care means

This theme identified that participants in general were unable to specifically describe what CDC actually meant, how it applied to them, and the rights and responsibilities of both themselves and their providers under the CDC model. Specifically, participants were unable to describe what constituted CDC services and what CDC services meant in a practical sense. Sometimes, this lack of knowledge was due to providers not informing the consumer sufficiently:

“Nobody tells you what is available to you” (Participant 1SE).

“When you first come in there should be more education for it somehow. I don’t know how but it can be very desperate for people before they get help” (Participant H03).

“I’d like to be told everything, you know…. More information…. ongoing information” (Participant H02).

Consumer knowledge around CDC was more from what the service provider could offer, rather than what the consumer might identify.

“Well it’s more the services that they could provide us with” (Participant 328).

“So she said, well it’s done by needs, so that if we just think you need it more then you’ll get it (service)” (Participant 4BP).

“…. they just suggested different things and I said yes” (Participant H02).

Most of those who had transferred from PDC services were unable to differentiate between their existing CDC service and their previous PDC service as Participant H02 describes: “I don’t know when the new program started, really, because I only read about it in the senior’s paper…. It’s just exactly the same as before (PDC)”.

Once a client had accepted a CDC service, they (or their carer) realised that they would need to become more actively involved in managing their service but found this challenging as Participant H04 described: “…. she explained all these things, you know, the weekly subsidy and weekly base care fees with the income test and all that…. that gets confusing”.

Knowledge and understanding of CDC in relation to the potential for a client to recognise and negotiate tailoring of services to meet specific needs was not a common finding:

“I didn’t know you could do that even… I don’t know, or have the experience to make the changes, you see, of what I would like to do” (Participant H04).

“I don’t see how I could have much more control over it” (Participant C04).

In fact, there was confusion for those who had changed from PDC, leading one client to question the benefits of the change:

“I had my son here as an advocate for me but then we still didn’t come to an understanding of what more I needed to pay on top of what I had already been paying for all these years. I mean it has gone up over the years admittedly, and that was fine, but then when you’ve got to stick to a particular budget and you want these services, I was having two hours cleaning a week, two hours shopping a week, I was having transport, if I needed to go to the doctor or the dentist or whatever. I was having massages six nights a week for my shoulders and upper back. Well, the bomb hit, my cleaning is now an hour and a half a week, my shopping is an hour and three quarters, I broke down the massage from six to three nights a week, and it still didn’t seem to be a satisfactory deal” (Participant 2SK).

A proactive approach by the service organisation was generally viewed as a positive by participants and unless they had previous experience or knowledge of service provision provided by other service organisations, participants usually chose the first service organisation to contact them. Many participants (12/14) indicated that they were happy with their provider and service.

Nevertheless, lack of specific understanding of how CDC actually worked could be influenced by ambiguity in knowing who to contact when questions arose as Participants 328 and H04 describe:

“The thing is, finding out exactly who I should talk to…. You know, if I want, like for instance, day trips” (Participant 328).

“I don’t know who’s our coordinator, you know? Who’s our care manager or whatever you call it. Nobody’s said anything about that so far” (Participant H04).

This also extended to being able to choose their preferred provider or understanding that they actually had a choice as Participants C03 and H01 identify:

“I don’t know exactly how to sort of get in touch with them (their preferred provider) and find out” (Participant C03).

“I never sort of think about those sort of things (different service)…. asking them” (Participant H01).

Almost every participant showed a lack of knowledge of the full range of services that could be made available whether it be the fact that they could change the services being received or have services not listed:

“But after the three months finished, I'm stuck, nobody come helping me…. My friend give me telephone number and she said: these people can look after you. Just ring and ask” (Participant C01).

“One thing that I would love to know, I didn’t ask (Care Worker), whether they have outings?” (Participant C03).

When conducting interviews, the researchers often found it hard to assess if the client had been offered services other than the standard services of personal care, transport, cleaning, gardening, shopping or assistance with paying bills which were activities regularly provided under PDC. This was made more challenging when participants who had previously received PDC demonstrated difficulty in their ability to remember or understand the actual service that they had previously received in comparison to their CDC service now. This may have been because many of the CDC clients had poor health with some having identified impaired cognitive ability to understand CDC or the services that could be provided as Participants H05 and 1SE described:

“I’ve sort of lost all my memory of what has happened. I can’t remember how I really got onto (the organisation)” (H05).

“…. my mind doesn’t take things in very well these days and when I think of all these jobs that I’ve had; I should understand everything but I don’t” (Participant 1SE).

Overall, clients were unable to describe what constituted a CDC service and what this meant to them in a practical sense. Importantly, as clients did not know or understand how CDC could benefit them from a person-centric perspective, they were unable to initially gain full benefit from their CDC allocation; that is, to choose a provider and services which best met their needs within the funding package they were allocated.

### Acceptance: happily taking any prescriptive service that is offered

This theme relates to participants accepting what was offered in their CDC service because they knew they needed assistance and were grateful to get anything they could. Participant C01 expressed this best: “I said thanks God it’s better than nothing when they come”.

Participants who were totally accepting of their service spoke very highly of their service provider and the help they received:

“it’s wonderful, wonderful. I’m very happy. They are very, very polite” (C02).

“I’m happy with what we’re doing at the moment. And everything is sort of going fine” (C03).

“she sort of lights my day up when she comes in …. she’s clean in her appearance, you know” (H05).

Others indicated that although the actual services provided might not be exactly what they would like, they were just pleased to be receiving it and were not looking for anything to change:

“I accept that (the way the service is provided) …. It’s better than the other alternative so you’ve got to accept as it is” (Participant H03).

“They’re (frontline workers) restricted in what they can do for me, but they vacuum and they do the bathroom well for me” (Participant H01).

“Well, I don’t ask for a lot see…. but there are lots of places you’re not alright and so they’re the things you don’t talk about so much” (Participant H01).

It became obvious that the CDC package provided a lifeline of independence and the ability for individuals to stay at home when family were not able to assist them to the extent they required:

“We had to get it (CDC service) in an emergency and they started straight away, you know, came as soon as we needed them and had to be assessed and all the rest of it has just been such a good help to us” (Participant H04).

“it’s taken a big burden off my shoulders, and a lot of anxiety trying to look for people to give me a hand and help me” (Participant C02).

Nevertheless, hesitancy in contacting service providers for different choices other than what was scheduled as other needs arose hindered the appropriate application of CDC. This resulted in clients merely accepting what was offered because this was seen to be more important than negotiating for a tailored CDC service, as the following participants tried to make clear without directly saying so:

“…. accepted everything they offered” (Participant C03).

“I’ve got no alternative, have I?” (Participant C04).

“…. so they can be late and things like that….Not their fault really, you know…. We’re quite happy with the times and things” (Participant H04).

Overall in spite of the challenges associated with the transition to CDC, these participants accepted and were grateful for the services they received and the way that they were helped by their service provider. However, this theme also depicts that as a consequence of their need for support, these participants were ready to accept whatever was on offer without questioning whether it met their specific and immediate needs.

### Compliance: unhappily acceding to the prescriptive service that is offered

This theme moves beyond the concept of acceptance to that of compliance with what the service provider offers rather than the client articulating or negotiating for a more personalised service because from their perspective they did not want to put the services or funding that they already had at risk. Several participants expressed this through the view that because service organisations and the services offered were underpinned by government regulations, they did not feel they could negotiate for more personalised services for fear of losing their current service, especially as they were aware that funding had to meet the needs of an overwhelming number of people. This can be seen in the expression of participant 2SK as they acknowledged: “Now we’re down to federal government allowing so much per client…. But I do know and I understand there is a waiting list, because they have so many people on their list and I appreciate that”.

In fact those who knew there were many other people on the waiting list were not willing to lose what they already had by pressuring for different services to what they were advised would be provided to them:

Participant H01 recounted “I understood what was going to happen, but I wasn’t happy about it” (as it was not the service they were wanting); and

Participant RH2SK indicated “I’m not happy with the situation …. that’s the way it’s got to be”.

To demonstrate a more detailed example of this passive compliance Participant H02 explained that “In the beginning they said they could be here between 9 and 1 and things like that and that didn’t suit me, you know, that really didn’t suit me. But I let it go in the end”.

Their expressed vulnerability was evident especially when participants explained they could not or would not rely on family support to assist them:

“My daughter would have stayed with me if I had wanted it but we didn’t want it. She’s in [an Asian country], but she’ll be here as fast as she could be. My son’s in [another state]” (Participant 4BP7).

“All my family is over in [another state]” (Participant RL03).

“My son is a grandfather now, he has his children, he has his grandchildren” (Participant 2SK).

“My son is supposed to do the outside and that and it’s up to him though, I can’t force him to do it, it’s up to him to do it…. I’ll tell you my son is 63 only a couple of weeks ago.” (Participant H01).

This need to comply for fear of losing what was offered prevented their pursuit of alternative services that they felt would have suited them better:

“We really didn’t like to ask because …. we never got any help before” (H03).

“Never asked. Well I don’t know whether they would do anything in the garden” (H04).

When asked to imagine other service possibilities H02 explained how they would like to “be told everything, changes they could make …. All that sort of thing” but didn’t feel they had sufficient understanding of CDC to know what to ask for.

Having to make such decisions and choices was acknowledged as difficult by those participants in more fragile health who may have been embarrassed about needing such assistance in the first place as Participant 1SE describes: “I’m afraid that I’m asking too much and their time spent taking up and they’re busy, I mean they have an awful lot of people (but) the only thing I would like to do is to get out more but I can’t without somebody with me, and I don’t like to ask too often to be taken out”.

The theme of Compliance differs in subtle and unique ways to the theme of Acceptance. Compliance is distinguished by these clients not being happy with aspects of their service, yet still complying even where they had identified that they wanted something different. In essence these clients complied because they did not want to jeopardise or lose their service especially as they understood the difficulties associated with getting support services and funding in the first place. This made these clients feel “hostage” to taking the proffered service which they acknowledged did not fully meeting their needs.

### External influences: previous aged care service experience, money availability and its management, and cultural differences

Participant H01 described how agency provider restrictions reduced the range of CDC services being made available: “(Service organisation) can’t do that for me, that’s not in their line”.

When clients were transferred to a CDC service from a PDC service, the result for some had been a change in the person providing their service leading to dissatisfaction with the new arrangements as Participant H01 describes: “The new system’s not as good …. the girls that were coming to me stopped…. they said it was partially the government had stepped in”.

Participants who had more money or prior PDC experience described how they got around restrictions or changes to their service as Participant H02 explains: “I pay the gardeners separate… I’ve had the bathroom remodelled, I’ve paid for that of course. And steps down the back, that’s all been remodelled so I have to pay for all that”.

To maintain their independence and get the best value for money from their newly created CDC services, clients with experience of PDC who were still cognitively competent seemed more proficient at getting the services they required. However financial stress was the more common theme:

“I am short with money, I do some knitting like socks and scarf and I sell it for $5. I buy the wool and I’m knitting…. I have plants, flowers…. I sell it for $5, $2 and I get some money to buy my medicine, to help me for bills to come, electricity comes, like this. I try to help myself” (Participant C01).

“They don’t realise how far that pension’s got to go…. I just couldn’t afford the difference (for additional service)” (Participant H01).

“They said would do it (mow the lawn) and the government would give me $20 off, but I couldn’t afford to pay the difference, it was out of the question” (Participant H01).

“I had to think about the money part of it. See, that’s the main thing was the money. See, I didn’t have the money to pay for it. So, you know, I don’t drink and I don’t smoke so, you know I gave up smoking years and years ago, because I couldn’t afford it” (Participant H05).

Participants 2SK and C01 described how they made their cash funds stretch as far as possible, even to the point of rearranging their CDC services to save money as well as giving up lifestyle choices that were costly:

“Now we’re down to federal government allowing so much per client…. My hours had to be cut because there wasn’t enough finance…. I eventually cut down to two sessions and she switched them round, she put the shopping girl on a Monday morning to clean, and put the cleaner on a late shop on a Friday, so that she could do some personal care for me before she left to save a night round fee and another trip which is time from A to B patients, and petrol, and night time round of costs” (Participant 2SK).

“They want money from me. I said, “I’m sorry, I don’t have money to pay you.” And I stopped because it was too much money to pay and I can’t do it.” (Participant C01).

Participants also described how they paid for other services that were not provided under CDC but which they felt they needed:

“When I moved I got the OT [occupational therapist] to come in and look around the place and just--she knew me well--saying what extra things would I need and she did them.” (Participant 4BP). “So was that OT service, did they come out of your CDC?” (Interviewer). “No I paid for that. Yes, I did pay for that because it’s through the health service, I get I think five things a year and I haven’t got any left and I felt I needed somebody to guide me”( Participant 4BP).

“The scooter’s just been repaired $600. And I had to have that repaired and I wasn’t able to use it, so I haven’t been going to the library” (Participant RH1SE). “So the scooter repair was that out of your own personal funds?” (Interviewer). “Yeah” (Participant RH1SE).

“So within the week that was two lots of taxi fares up and back so it was $20 gone. Sometimes it can be $30 it depends where I’m going” (Participant RH2SK).

Other participants weren’t clear who was providing which services but they knew how much in extra payments for non-CDC services they were willing to pay as Participant H01 explains: “Yes some comes from the Baptist Church, I don’t know who informed them, but they came or they sent me a letter offering me to do the lawn mowing and that, because the grass was like that at the time, very high. And they said they would do it and the government would give me $20 off, but I couldn’t afford to pay the difference, it was out of the question”.

Participant H03 was aware of additional lifestyle choices being provided outside of the CDC structure: “If we want to go on a trip with them there’s a place down here where you can go with them (another organisation). They take you for outings and all that and it costs you about $15 which is not bad”.

The impact of cultural differences on participation and comprehension was evident:

“I went once to X Group but…. some were from other countries and if they had a couple from their own country they would talk amongst themselves in their own language that I couldn’t understand and the other ladies couldn’t understand. So it was a sort of a segregated group so I didn’t like it” (Participant H02).

Participants whose first language was not English explained their difficulties with much of the documentation and reports they were given which were in English:

“I no read English…. ” (Participant C01).

“I got my daughter-in-law and my daughter to take over that reading” (Participant C02).

In summary, this theme identifies that with the current roll out of CDC the ability to adequately compare the experiences of participants receiving the current version of CDC is limited, in part due to external factors, such as finances, culture and previous experience influencing the way the packages are being used. Nevertheless, these results provide rich detail about the positive and negative attributes of the current manner in which CDC is being implemented from the consumer perspective.

## Discussion

To understand the CDC experience from the consumer’s perspective, our study explored how the person receiving a CDC package learned about CDC; their understanding of how it worked; what they thought were the range and type of service possibilities for them; whether the services they received met their needs; their financial understanding of their CDC service; as well as any additional costs incurred.

Consumer Directed Care has as its central objective to maintain or improve the quality of life (QOL) of older people, by supporting them to not just remain living in their own home but also to continue to enjoy living there [14]. Lawton’s [26] multidimensional conceptualisation of QOL in old age identified that QOL dimensions occur on an objective-subjective continuum with circumstances and the environment influencing the meanings a person applies to assess their QOL. Our study found that factors external to the immediate physical needs of the individual were equally as important to their overall sense of wellbeing as more direct factors supporting their physical needs. For example, maintaining the garden and day trips were just as important for their QOL as having help dressing, shopping or paying bills. This is consistent with Lawton [26] who reported that QOL was ultimately determined by the individual’s psychological well-being. In our study, participant psychological wellbeing included social and family dynamics as well as acceptance of their need for assistance.

Thomas and Browning [27] identified that social connectedness, mastery and autonomy are important determinants of a person’s QOL and that these are achieved through the delivery of a service that the person wants rather than what a provider may think is needed. For this to be achieved, support is needed to facilitate the level of philosophical and cultural change required to achieving a consumer led service which should then result in a client receiving the service they want [27]. Our study indicates that when consumers are supported to make decisions that reflect their needs within the confines of their CDC package, they are able to better negotiate and use multiple sources of support to maintain their independence and social engagement.

The recent literature highlights that access to information is fundamental to a person aging with dignity to maintain their independence [13] but clients must have the cognitive ability, support and confidence to know where to look for and gain access to information about the variety of services available to assist them to remain independently in their own home [28, 29]. Coming to terms with needing assistance and the cognitive ability to manage decisions about service provision as well as being able to access what is needed are elements of CDC provision that need to be seen as core elements for CDC to succeed. Vik an Eide [30] identified that if this is not done, clients may be unlikely to request the real services they need until they receive active encouragement and support.

Bowling et al. [31] found that the non-physical dimensions of care were increasingly nominated by community-dwelling older people as being an important aspect of improving their QOL. Bulamu et al. [20] found for people receiving CDC services where these non-physical dimensions of care were addressed, higher levels of independence and control were reported.

Clients appeared to rationalise the provision of the services they received, based on their appreciation of the difficulties being experienced by service providers were constrained by legislative schedules that defined the range and scope of services that could be provided at each package level whilst also striving to transition to CDC whilst meeting the needs of an expanding client base. However, the reality for many of the participants was that the services they received did not sufficiently meet all of their needs in the way that was most helpful to them. Clients therefore had to compromise and make trade-offs, often on a weekly or monthly basis, but their ability to effectively do so was related to their state of health or their partner’s state of health at that time. This is an important finding for service providers especially with regard to the training of frontline staff to ensure these staff can identify and report when clients have become unable to self-manage their CDC. At this point the service agency can then make recommendations to provide greater assistance. Nonetheless, any changes to the use of client funds and their service plan requires the client’s or their advocate’s consent including whether the client self manages their package or whether they agree to pay for the provider to do this on their behalf. Knowing when a person/family is not managing well and the optimal time to step in without appearing to take over decision-making may pose some challenges for providers.

Our findings suggest that to empower consumers to manage their CDC service in a way that more appropriately meets their needs, simplified language and repetition of information with explanatory communication, both written and verbal, would be helpful. This may be especially true for clients with reduced cognitive ability or clients from a CALD (culturally and linguistically diverse) background or with lower levels of literacy where the ability to understand written information may be diminished [32]. Further, the findings from our study concur with other recent evidence to suggest that if clients are struggling with engagement in CDC service provision and management, a more managed approach over a longer period of time may be needed until the clients are more conversant with how CDC services work, what choices they have as well as who and how to contact the service provider when needs change [33, 34].

Not having sufficient understanding of the range of CDC provisions, services and activities led participants in this study to suggest ways in which they could be better assisted to gain a more thorough understanding of CDC. Participants indicated that they would like a list of all the available organisations providing CDC services in their area as well as to know how the organisation that had contacted them had learnt about their needs. They also pointed out that they would like an explanation of how their service manager had been chosen along with an engagement process that includes follow-up assessments and when they are to be conducted, why and under what conditions their level of service will be determined (this may need to be done a number of times at the beginning of the service while the person gets used to CDC). In addition, participants in this study suggested that they would like assistance to better understand the breakdown of their CDC budget, including all administrative charges, so they can better determine how their allocation can be utilised within the conditions that apply.

### Limitations

This research was conducted prior to the full implementation of the Australian CDC program, so the results from this study are possibly both context and time limited. It was also conducted on a relatively small sample of participants (14) drawn from only five service provider organisations in two states. However, in qualitative research data saturation indicates that when no new knowledge is forthcoming, the number of participants is sufficient to answer the research question posed.

Participants self-selected to participate which could have created a selection bias. The expansion of the recruitment pool to include clients who had transferred from PDC to CDC may have influenced the findings due to their prior experience of that different service model. It did not include participants living in non-urban areas and it did not assess the effectiveness of CDC execution by the service provider organisations. Future research should involve more provider organisations and different levels of package recipients, as well as inclusion of people living in non-urban areas as well as observation of participants who have had CDC packages for longer than 12 months to understand whether, once they gain familiarity with the program, they become more proactive in changing services and service providers to meet their needs. It is also important to compare different organisation models and intra-organisation assessments. This will facilitate the identification and mapping of the changes that have occurred over time and allow the program’s impact and effectiveness to be evaluated more fully.

## Conclusions

Our findings are consistent with other investigations that have identified a number of areas of concern to consumers receiving home care services (CDC) including: costs related to administrative fees and brokerage; issues with accuracy and complexity of information; limited choice and flexibility in the service and who provides it; issues with goals not being reflected in care plans; and inequities in access and service availability. The process of identifying and developing service options must be collaborative, given the ultimate aim is for services that are client specific, directly relevant and acceptable to them. Ongoing engagement and discussion with clients is then necessary to evaluate the client’s view of their service’s effectiveness, including how it meets their needs and impacts on their QOL and health.

Whilst the issues identified in this paper are not unexpected for a policy change in its infancy in Australia, the uniqueness of this study is that it provides some insight into the management and use of CDC services from a CDC client perspective rather than a policy or service provider perspective. Our findings therefore suggest that to support the progressive roll-out of CDC over the next few years, more intensive efforts will be needed to inform, engage and assess how the service provided matches the needs of the individual and maintains or improves their QOL. Importantly, providing non-physical or non-traditional services to CDC clients requires service managers to take time to review the client on a regular basis and to act on requests and feedback via frontline staff, so as to empower the client to self-determine their service needs. It may not always be prudent for service organisations to depend on the client contacting the service manager for nominating specific services as clients may be unaware or lack the confidence in asking for all of the service possibilities available or more suitable to them.

The transition to CDC will no doubt be labour and administratively intensive at first until CDC becomes a normalised function of aged care service provision. However, this should not deter agencies and government funders from resourcing this intensity in the first few years of CDC. Overtime, as this model matures, government will need to invest further in making sure the community is aware of how this program works and what a client can reasonably expect to negotiate. As more and more people choose to remain at home as long as possible, the idea that people will move into residential aged care to have their needs met becomes neither reasonable nor sustainable. Consumer directed care offers a means to ensure people remain at home, independent and functioning well for as long as possible. For this program not to succeed, will mean a failure on society’s part to ensure our future choices as individuals.

The future research needed to improve understanding of how a CDC service can better achieve its objective of meeting specific client needs includes:

- Investigating frontline worker feedback of client articulated needs and comparing this to the service design process
- Exploring implementation of a system of specified and regular contact with the client, especially during the first six months of CDC package implementation, to assess whether the services are meeting client specific need
- Investigating the active provision of a range of alternative, non-physical types of services based on client led discussion and measuring QOL over time
- Regular assessment of the costs and financial considerations of different services offered and how these could be better managed within the financial constraints of the service and the resources of the individual
- Investigating how well CDC services are being executed
- Observing the support mechanisms in place to enable clients to transfer to another provider where the desired service cannot be sourced with their current provider
- Understanding what type of decision-supports and assistance are needed for clients to self-manage their CDC package so that they are able to identify their needs and choose services and levels of service that best meet their requirements
- Long-term monitoring of the cognitive status of the clients in receipt of CDC packages and whether there needs to be differentiation of servicing based on the client’s cognitive level

## Additional files

Additional file 1: Qualitative Interview Question Guide. (PDF 54 kb)

Additional file 2: CDC Codes and Categories. (PDF 283 kb)

## Abbreviations

CDC: Consumer Directed Care
NSW: New South Wales
OT: Occupational therapist
PDC: Provider Directed Care
QOL: Quality of life
SA: South Australia
