# Supplementary material for: How do clients in Australia experience Consumer Directed Care?
Source: BMC Geriatr. 2018 Jun 26;18:148. doi: 10.1186/s12877-018-0838-8 (PMC6019791; doi:10.1186/s12877-018-0838-8)
Supplement: Supplementary file 1 — Qualitative Interview Question Guide. (PDF 54 kb) [file 12877_2018_838_MOESM1_ESM.pdf]

Additional file 1

Qualitative Interview Question Guide

1. Can you tell me how (organisation xxx) introduced CDC to you…..
   - who first introduced CDC to you,
   - what information were you given
   - who gave this information to you,
   - in what form was the information provided (verbal/written etc) and
   - were you given any suggestions or assistance to help you make your decision of the service you would like to receive? Please explain.
2. Can you tell me what you understand makes a service Consumer Directed?
3. What role did you play in determining the actual service/s you are receiving?
4. Can you tell me how much control you felt you actually had in determining your actual service?
   - would you have liked to have had a different amount of control in determining your service?
   - same/more/less? (Ask for elaboration)
5. From your perspective have you experienced any issues with regard to your services?
   - changes (increase or decrease)
   - restrictions/limits (time, day etc)
   - issues arising due to the people providing your service being employed by a different organization to those who are managing your CDC service
   - additional costs
   - unanswered questions
   - out of pocket expenses
   - any other

6. Are there any support services you feel you need but you have not been able to get under CDC?

   – if yes, what do you think is preventing you getting these services? How do you know this?

7. Are you currently paying for any additional support services that are not able to be provided to you under your CDC service? If yes please explain.

8. Have you taken up all of the CDC services you have been offered?

   – if not, what were the services that you decided not to have?…..can you explain your reasons?

9. Have you personally asked for any particular service that you feel you need?

   – if yes ….. can you tell me what has happened as a result of your request?

10. Is there any service you would like to receive but so far have not asked for?

    – if yes …….can you tell me what it is that you would like to receive?

    – why haven't you asked for it?

11. Have you ever received any reports or statements from organisation xxxx about your CDC service?

    – if yes …….can you tell me about what you have received?

    – was it useful?.....please explain.

12. Have you experienced any problems or limits with your CDC services?

    – if yes …….can you tell me about your experience?

13. Can you tell me what you feel the overall impact of CDC services has been on you?

    – its results, any changes, any benefits, and disadvantages and so on?

14. Do you have any suggestions as to how CDC services might be improved?

15. Would you be likely to recommend CDC services to your friends or other people?

    –can you tell me why or why not?
