# Supplementary material for: How do clients in Australia experience Consumer Directed Care?
Source: BMC Geriatr. 2018 Jun 26;18:148. doi: 10.1186/s12877-018-0838-8 (PMC6019791; doi:10.1186/s12877-018-0838-8)
Supplement: Supplementary file 2 — CDC Codes and Categories. (PDF 283 kb) [file 12877_2018_838_MOESM2_ESM.pdf]

Additional file 2: CDC Codes and Categories

Code Number	Code Name	Associated Category	Categories	Code Number	Associated Themes	Category Numbers for Themes
1	After Effects	2	Getting Information rather than knowledge	1	Not Sure What CDC is	1,5,7,9
2	Agency Identifiers	1,7	Accepting Compromises and Changes to Current Service Provision	2	I am grateful for the services provided to me	2,6,8
3	Banking	2	Rationalising the Quality of Care	3	Without these services I would not be able to do what I do	3,4
4	Care Provision	9	Non-consideration of alternatives	4		
5	CDC System	1	Lack of understanding of terminology and information in reports	5		
6	Change	4	Vulnerability to Service Reduction	6		
7	Choice of Carer	7	Administration	7		
8	Choice of CDC Package Manager	7,9	Experience with previous aged care packages	8		
9	Communication with Others about CDC	8	Assessment Process	9		
10	Compliance by Agency	2,8				
11	Confidence	6				
12	Confidentiality	7				
13	Confirmation of Knowledge	1,8				
14	Control of Service	2,7				
15	Cost	3				
16	Day Care	2				
17	Discussion	1,8				
18	Disease State	6				
19	Dissatisfaction	2,9,6				
20	Distance	2				
21	Dying and Death	6				
22	Emotional Response	6				
23	Establishment of Service	1,7,9				
24	Expectations	3				
25	Extension of Care	2,3,4,6				
26	Family Care Provision	2,3,4,6				
27	Food Preparation	2,4				
28	Funerals	3				
29	Future Recommendations	2,3				
30	Gardening	2				
31	Gender of Personnel	2,3				
32	Government	7,8				
33	House Maintenance	2				
34	Incontinence	6				
35	Just Told	1,8,2				
36	Length of time of service of individual carers	2				
37	Limits to preferred services	2,3,8				

38	No choice in personnel	2				
39	No choice in time	2				
40	No Knowledge	1,5,8				
41	Non-recognition of Previous Carers	2				
42	Outside Institutions	2,4				
43	Paying Bills	2,7				
44	Physical Disabilities	6				
45	Places of Care	2,4				
46	Podiatry	2				
47	Reason for Type of Care	2,6				
48	Religion	3				
49	Satisfaction	8				
50	Service Use Prior to CDC	2,8				
51	Services Not Provided	2,4				
52	Setting Preferences	2,3,4,8				
53	Shopping	2				
54	Social Activities	2				
55	Social Pressures	2,3,4,6,8				
56	Statements	5				
57	Terminology	5,7				
58	Time	2,3,4,6,7,8				
59	Time After Initial CDC Care	8				
60	Travel	2				
61	Types of Service	2				
62	What CDC Means	1,8				
63	When Service First Started	1,8,9				
64	Who Told Client About the Program	1,7,9				
65	Widowhood	6				
